# Commentary on ‘Data-driven coaching to improve statewide outcomes in CABG: before and after interventional study’

**DOI:** 10.1097/MS9.0000000000002467

**Published:** 2024-08-22

**Authors:** Qingping Xia, Chengbin Zhou, Li Deng

**Affiliations:** a Department of Science and Education, The People’s Hospital of Gaozhou, Gaozhou; b Department of Extracorporeal Circulation, Guangdong Provincial People’s Hospital, Guangdong Academy of Medical Sciences, Guangzhou; c Department of Cardiovascular Surgery, Affiliated Gaozhou People’s Hospital, Guangdong Medical University, Gaozhou, Guangdong, People’s Republic of China

*Dear Editor*,

I am writing to offer my commentary on the recent study titled ‘Data-driven Coaching to Improve Statewide Outcomes in CABG: Before and After Interventional Study’ in the International Journal of Surgery^1^. This study provides valuable insights into the potential of data-driven quality improvement plans (QIPs) in enhancing the outcomes of coronary artery bypass grafting (CABG) surgeries. I commend the research team led by Omar A. V. Mejia for their innovative work.

## Background and significance

CABG, while a life-saving procedure, has historically struggled to improve surgical quality and reduce postoperative complications. The authors of this study argue that traditional approaches, which often focus solely on technical skills, are insufficient^2^. They propose a QIP incorporating nontechnical skills training and surgical coaching as a potential solution. This concept is significant given the lack of a nationally standardized and data-driven QIP that can be readily scaled and replicated across centers.

## Methodology and findings

The prospective cohort study utilized data from the multicenter database, Registro Paulista de Cirurgia Cardiovascular II (REPLICCAR II), covering a 2-year period from July 2017 to June 2019. Analyzing data from 4018 adult patients who underwent isolated CABG surgeries, the study aimed to demonstrate the effectiveness of QIP measures in reducing mortality and complications. The findings indicate that nontechnical skills training and surgical guidance played a crucial role in reducing postoperative failures, thus leading to fewer rescues and significant reductions in mortality rates and complications such as deep sternal wound infections, sepsis, and prolonged ventilation times.

## Impact and limitations

The study’s findings are highly significant, demonstrating the power of combining nontechnical skills training, surgical coaching, and data-driven insights to improve surgical outcomes. This approach not only enhances surgical quality but also empowers surgical teams to proactively identify and address issues, ultimately leading to better patient care.

However, the study has several limitations. Firstly, the QIP did not incorporate risk stratification using widely accepted tools like EuroSCORE II, which could provide a more nuanced understanding of its effectiveness across different patient populations^3^. Secondly, there is a lack of explicit quantitative metric specifications for components like glycemic control and blood management, which may hinder the ability of readers to replicate the QIP model. Achieving a uniform standard across centers can be challenging due to inherent differences in circumstances.

## Recommendations for future research and QIP enhancement

To further enhance the QIP and address some of its limitations, I recommend several areas of focus. Firstly, given that numerous studies have shown that nutritional and inflammatory status are significant predictors of mortality in cardiac surgery patients^4^, the QIP should be expanded to include the management of patients’ nutritional and inflammatory profiles. This would enrich the QIP’s metrics and enhance its comprehensiveness and efficacy in improving CABG outcomes. Secondly, future QIPs should be formulated with more reference to international and domestic authoritative guidelines, consensus statements, and best practices. Explicitly outlining quantifiable indicators and reference ranges for key components such as glycemic control and blood management will provide implementers with more specific operational guidance and facilitate data comparisons and experience sharing between centers. Thirdly, a randomized controlled trial would provide a more rigorous assessment of the effectiveness of the data-driven coaching program. A multicenter study with a larger sample size would also allow for a more comprehensive evaluation of the program’s implementation and outcomes across different settings. Finally, future research could explore the impact of patient-specific factors, such as comorbidities and surgical complexity, on the effectiveness of the QIP. Understanding these factors could help refine the QIP and ensure its optimal performance across diverse patient populations.

## New metric and potential for further exploration

The study introduces the concept of ‘rescue failure’ as a new metric to assess surgical team performance, highlighting the importance of proactive management and prevention of complications. Future research could further explore the factors contributing to rescue failure and strategies to mitigate its occurrence.

## Conclusion

In conclusion, the study represents a significant step forward in surgical quality improvement, demonstrating the potential of data-driven coaching programs in improving CABG outcomes. While further research is needed to address limitations, the findings provide a strong foundation for the continued development and implementation of such programs in surgical care. Thank you for considering my commentary. I look forward to discussing this important work further.

## Ethical approval

Not applicable.

## Consent

Not applicable.

## Source of funding

This study was supported by grants from the Guangdong Province's major Heart Disease Diagnosis and Treatment Engineering Technology Research Center program (2017).

## Author contribution

Q.X.: drafted the manuscript; L.D.: conceived important content and approved the manuscript; C.b.Z.: revised the manuscript critically for important intellectual content.

## Conflicts of interest disclosure

The authors declare no conflicts of interest.

## Research registration unique identifying number (UIN)

Not applicable.

## Guarantor

Li Deng.

## Data availability statement

Not applicable.

## Provenance and peer review

Not applicable.
